# Evolutionary Pattern of the *FAE1* Gene in Brassicaceae and Its Correlation with the Erucic Acid Trait

**DOI:** 10.1371/journal.pone.0083535

**Published:** 2013-12-16

**Authors:** Xiaoqin Sun, Hui Pang, Mimi Li, Bin Peng, Haisong Guo, Qinqin Yan, Yueyu Hang

**Affiliations:** Jiangsu Province Key Laboratory for Plant Ex Situ Conservation, Institute of Botany, Jiangsu Province and Chinese Academy of Sciences, Nanjing, Jiangsu, China; Durham University, United Kingdom

## Abstract

The *fatty acid elongase* 1 (*FAE1*) gene catalyzes the initial condensation step in the elongation pathway of VLCFA (very long chain fatty acid) biosynthesis and is thus a key gene in erucic acid biosynthesis. Based on a worldwide collection of 62 accessions representing 14 tribes, 31 genera, 51 species, 4 subspecies and 7 varieties, we conducted a phylogenetic reconstruction and correlation analysis between genetic variations in the *FAE1* gene and the erucic acid trait, attempting to gain insight into the evolutionary patterns and the correlations between genetic variations in *FAE1* and trait variations. The five clear, deeply diverged clades detected in the phylogenetic reconstruction are largely congruent with a previous multiple gene-derived phylogeny. The Ka/Ks ratio (<1) and overall low level of nucleotide diversity in the *FAE1* gene suggest that purifying selection is the major evolutionary force acting on this gene. Sequence variations in *FAE1* show a strong correlation with the content of erucic acid in seeds, suggesting a causal link between the two. Furthermore, we detected 16 mutations that were fixed between the low and high phenotypes of the *FAE1* gene, which constitute candidate active sites in this gene for altering the content of erucic acid in seeds. Our findings begin to shed light on the evolutionary pattern of this important gene and represent the first step in elucidating how the sequence variations impact the production of erucic acid in plants.

## Introduction

Erucic acid is one of the major fatty acids present in the oil extracted from members of the family Brassicaceae. Together with other very long-chain fatty acids (VLCFAs; >18C), erucic acid is a precursor of many biologically important compounds, such as waxes [1], sphingolipids [2,3] and triacylglycerols [4]. In *Arabidopsis*, *FAE1* encodes for a b-ketoacyl-CoA synthase (*FAE1* KCS) that catalyzes the initial condensation step in the elongation pathway of VLCFA biosynthesis [5]. 

After the first *FAE1* gene was cloned in *Arabidopsis* via transposon tagging [5], Lassner et al. [6] cloned a cDNA homologous to the *Arabidopsis FAE1* gene from jojoba and the role of this cDNA in producing erucic acid was genetically ascertained through genetic transformation of low erucic acid content rapeseed. In addition, two loci, E1 and E2, encoding KCS enzymes were isolated from rapeseed using an oligonucleotide probe based on the *Arabidopsis FAE1* gene [7,8]. In *Brassica napus*, two homologs of the *FAE1* gene (Bn-*FAE1*.*1* and Bn-*FAE1*.*2*) have been characterized, showing 99.4% nucleotide identity and a two-base deletion resulting in functional loss of the Bn-*FAE1*.*2* gene in the C genome [8]. The LEA trait of rapeseed of the ORO origin is attributed to the substitution of a single amino acid residue, replacing serine with phenylalanine at position 282 of the encoded protein [9,10]. Based on the alignment of amino acid sequences, the putative active site of the KCS, Cys223, His391 and Asn424 were predicted to be crucial for enzyme function [11,12]. These amino acids, together with four conserved histidine residues and six conserved cysteine residues identified by Ghanevati and Jaworski [11], were subsequently found to be present in all of the HEA and LEA rapeseed cultivars analyzed to date [9,10,13]. Thus, the impact of sequence variations on the content of erucic acid in the seeds of Brassicaceae remains unclear. In addition, non-3 integer deletions in a fragment of the *FAE1* gene, which are predicted to lead to frameshift mutations and premature termination of translation, were shown to be responsible for the low erucic acid trait in rapeseed independent from the point mutation [8,14]. 

Although *FAE1* genes from different Brassicaceae species have been isolated and manipulated extensively through expression in homologous species or in heterologous host systems, the evolutionary history of *FAE1* in Brassicaceae remains unclear, and few studies have been focused on oil crops or the model plant species. For example, based on analysis of the evolutionary relationships and sequence similarities among three *Brassica* species, a closer pedigree relationship was identified between the *Brassica* A and C genomes than between the A/C genomes and the *Arabidopsis* genome. Additionally, 18 SNPs have been found in the coding region of *FAE1* that may be used as genome-specific markers to differentiate the A and C genomes [15]. Cloning and phylogenetic analyses of *FAE1* in *Camelina sativa* support a history of polyploidization and indicate that *C. sativa* and *C. microcarpa* might share a parental genome [16]. 

Up to 338 genera and 3,709 species are included in Brassicaceae [17]. However, the taxonomy of Brassicaceae has long been controversial because of the often poorly delimited generic boundaries and artificially circumscribed tribes, resulting in a lack of agreement with regard to the number of and boundaries between tribes and genera, giving rise to several systems of classification put forth during the past two centuries [18]. Recently, a comprehensive study was performed to assign 301 genera (94%) of the family to 49 tribes, suggesting an updated framework for the entire family [19]. Based on the combination of molecular work with trichome characteristics, three major lineages have been proposed during the last decade in the core Brassicaceae, which are sister to the basal group (*Aethionema*) [20]. These lineages are well supported by multiple plastid and nuclear sequences, including *phy*A [21], ITS [22], *nad*4 intron 1 [23] and combined molecular datasets (*adh*, *chs*, ITS, *mat*K, *trn*L-F [24] and *adh*, *chs*, ITS, *mat*K, *nad*4 intron 1, *ndh*F, *rbc*L, *trn*L-F [25]).

The content of erucic acid in the seeds of Brassicaceae is genetically variable [26,27]. As the *FAE1* gene serves as the key regulator in erucic acid biosynthesis, an evaluation of the diversity and a phylogenetic analysis of the *FAE1* gene were performed in this study to elucidate how the plant *FAE1* gene originated and evolved. Detection of variations and a correlation analysis between the *FAE1* genotype and phenotype were also performed to demonstrate how these genetic variations impact the erucic acid content in plants.

## Results

### *FAE1* Polymorphism

Forty-eight accessions were collected for *FAE1* cloning from around the world (the sequences of these accessions are provided with the Genbank accession number of initial letters "KF" in Table 1). Orthologous sequences of *FAE1* from *Arabidopsis thaliana*, *Arabidopsis lyrata* subsp. *kamchatica*, *Brassica napus*, *Brassica rapa*, *Brassica oleracea*, *Crambe glabrata*, *Crambe kralikii*, *Crambe hispanica* subsp. *abyssinica*, *Isatis tinctoria*, *Lepidium apetalum*, *Orychophragmus violaceu, Sinapis alba*, *Sinapis arvensis* and *Teesdalia nudicaulis* were downloaded from GenBank (the GenBank accession numbers are provided in Table 1). In total, 62 accessions, representing 14 tribes, 31 genera, 51 species, 4 subspecies and 7 varieties were used in this study. Among these accessions, the entire coding region of *FAE1* could be assessed in 26 accessions, whereas only partial fragments could be obtained in the other 36 accessions due to failure of PCR amplification with all four published sets of primers [14,28-30]. All 96 obtained sequences share high homology with the *FAE1* gene of *Arabidopsis thaliana* (GenBank accession: U29142.1), showing an identity higher than 80%. Among the 11 accessions randomly selected for a heterozygosity analysis of the *FAE1* gene, 7 accessions harbor different *FAE1*copies numbers, ranging from one copy in *Brassica tournefortii*, *Eruca vesicaria* subsp. *sativa*, *Coronopus didymus* and *Thlaspi arvense* to three in *Crambe hispanica*. This observation suggested that the gene may have undergone copy number evolution. The length of the alignment matrix is 892 bp, corresponding to 431~1,322 bp in the *FAE1* protein-coding region beginning with the start codon of *Arabidopsis thaliana* (GenBank accession: U29142.1), which contains no introns. A total of 489 variable sites and 375 informative sites were found, accounting for 54.82% and 42.04% of the matrix, respectively. 

**Table 1:** Collection of Brassicaceae species for evolutionary analysis of the *FAE1* gene.

Tribe	Genus	Species	Depositary (Accession); Geographical Origin	Genebank No.*
Lepidieae	*Lepidium*	*L. campestre* (L.) R. Brown	-	FJ907545.1
		*L. apetalum* Willdenow	GRIN (PI633246); Mongolia	KF030527
	*Coronopus*	*C. didymus* (L.) Smith	Nanjing Botanic Garden Mem. Sun Yat-Sen. (76A); China	KF664174
	*Cardaria*	*C. draba* (L.) Desvaux	Xinjiang Agricultural University (41A); China	KF030548
		*C. draba* subsp.*chalepensis* (L.) O. E. Schulz	Xinjiang Agricultural University (41B); China	KF030549
Cardamineae	*Rorippa*	*R. indica* (L.) Hiern	Nanjing Botanic Garden Mem. Sun Yat-Sen. (18A); China	KF030537
		*R. dubia* (Persoon) H. Hara	Nanjing Botanic Garden Mem. Sun Yat-Sen. (18B); China	KF030538
	*Nasturtium*	*N. officinale* R.Brown	Germplasm bank of wildspecies (59A), SW China; China	KF030552
	*Cardamine*	*C. hirsuta* L.	Nanjing Botanic Garden Mem. Sun Yat-Sen. (16A); China	KF030533
		*C. parviflora* L.	Xinjiang Agricultural University (16B); China	KF664172, KF664173
Camelineae	*Camelina*	*C. sativa* (L.) Crantz	GRIN (PI258366); Former Soviet Union	KF030544
		*C. microcarpa* de Candolle	GRIN (PI633186); Hungary	KF030545
	*Neslia*	*N. paniculata* (L.) Desvaux	PGRC (CN105426); Canada	KF030532
	*Capsella*	*C. bursa-pastoris* (L.) Medikus	Nanjing Botanic Garden Mem. Sun Yat-Sen. (11A); China	KF664170, KF664171
	*Arabidopsis*	*A. thaliana* (L.) Heynhold	-	U29142.1
		*A. lyrata* subsp. *kamchatica* (L.) O’Kane & Al-Shehbaz	-	GU929425.1
Erysimeae	*Erysimum*	*E. siliculosa* (M. Bieb.) Andrz.	Xinjiang Agricultural University (52A); China	KF030551
		*E. cheiranthoides* L.	GRIN (Ames23897); Germany	KF030541
		*E. sisymbrioides* C. A. Meyer	GRIN (Ames24353); Pakistan	KF030542
	*Cheiranthus*	*C. cheiri* L.	The Bordeaux Botanical Garden (0247SU2010); France	KF030557
Descurainieae	*Descurainia*	*D. sophia* (L.) Webb ex Prantl	PGRC (CN105424); Canada	KF030546
Brassiceae	*Brassica*	*B. oleracea* L.	-	GU325726.1-GU325732.1
		*B. oleracea* var. *botrytis* L.	GRIN (PI115881); India	KF030504
		*B. oleracea* var. *italica* Plenck	GRIN (PI231210); Italy	KF030505
		*B. oleracea* var. *gemmifera* (de Candolle) Zenker	GRIN (PI209942); Australia	KF030506
		*B. oleracea* var. *gongylodes* L	GRIN (PI211908); Iran	KF030507
		*B. oleracea* var. *albiflora* Kuntze	GRIN (PI249556); Thailand	KF030508
		*B. rapa* L.	-	GU325723.1-GU325725.1
		*B. nigra* (L.) W. D. J. Koch	PGRC (CN31735); Netherlands	KF030510
		*B. juncea* (L.) Czernajew	GRIN (Ames15645); America	KF030511
		*B. napus* L.	-	U50771.1, AF009563.1, AY888037.1, AY888043.1, AY888044.1, EU131166.1, EU543282.1, EU543283.1, GU325717.1-GU325722.1
		*B. napus* var. *napobrassica* (L.) Reichenbach	GRIN (PI263056); Russian Federation	KF030512
		*B. elongata* Ehrhart	GRIN (Ames21299); Iran	KF664168, KF664169
		*B. carinata* A. Braun	PGRC (CN40216); Canada	KF664166, KF664167
		*B. tournefortii* Gouan	PGRC (CN43445); India	KF664165
	*Sinapis*	*S. alba* L.	-	AY888040.1
		*S. arvensis* L.	-	AY888041.1, FJ870905.1
	*Diplotaxis*	*D. muralis* L.	PGRC (CN102087); Canada	KF030518
		*D. tenuisiliqua* Delile	PGRC (CN105425); Canada	KF030519
	*Eruca*	*E. vesicaria* subsp.*sativa* (Miller) Thellung	GRIN (Ames23027); Poland	KF664156
	*Raphanus*	*R. sativus* L.	GRIN (PI109146); Turkey	KF030521
		*R. raphanistrum* L.	GRIN (PI271451); India	KF664162, KF664163
	*Crambe*	*C. hispanica* *subsp* *abyssinica* Hochst. ex R. E. FrFr.	-	KC565738.1- KC565742.1
		*C. hispanica* L.	GRIN (Ames23114); Spain	KF664157-KF664159
		*C. glabrata* DC.	-	KC565749.1
		*C. kralikii* Coss.	-	KC565748.1
		*C. filiformis* Jacq.	PGRC (CN102050); Canada	KF664160, KF664161
	*Erucastrum*	*E. canariense* Webb&Berthel	PGRC (CN51419); Canada	KF030547
		*E. gallicum* O.E.Schulz	PGRC (CN102119); Canada	KF030559
	*Orychophragmus*	*O. violaceus* (L.) O. E. Schulz	-	AY888042.1
Isatideae	*Isatis*	*I. tinctoria* L.	-	AY888038.1
	*Myagrum*	*M. perfoliatum* L.	Zurich Botanic Garden (19964884); Switzerland	KF030555
Sisymbrieae	*Sisymbrium*	*S. officinale* (L.) Scopoli	PGRC (CN102228); Canada	KF030543
		*S. loeselii* L.	Xinjiang Agricultural University (50A); China	KF030550
Chorisporeae	*Chorispora*	*C. tenella* (Pallas) de Candolle	GRIN (PI650169); Iran	KF030539
Thlaspideae	*Thlaspi*	*T. arvense* L.	GRIN (Ames22461); Poland	KF664164
		*T. perfoliatum* L.	GRIN (Ames22569); Germany	KF030530
Alysseae	*Lobularia*	*L. maritima* (L.) Desv. var. *benthamii* (Voss.) L.H. & E.Z.Bailey.	Berlin Botanical Garden (1994601); German	KF030558
Calepineae	*Goldbachia*	*G. laevigata* (Marschall von Bieberstein) de Candolle	GRIN (Ames21365); Iran	KF030540
Iberideae	*Teesdalia*	*Teesdalia nudicaulis* (L.) R. Br.	-	EF186003.1
Aethionemeae	*Aethionema*	*A. grandiflorum* Boiss. & Hohen.	Berlin Botanical Garden (BONN2018); German	KF030553
unplaced	*Lunaria*	*L. annua* L.	Zurich Botanic Garden (55412005); Switzerland	KF030554

^*^ The sequences with the Genbank No. of initial letters "KF" were sequenced by ourselves and the remaining sequences of 14 species were downloaded from Genbank.

The nucleotide diversity (Pi) increased to 0.1024 and 0.0994 when *Aethionema grandiflorum* was excluded. The pairwise distances of the *FAE1* sequences among ingroup taxa ranged from 0, between 97 pairs of species in Brassiceae and one species pair each in *Rorippa* and *Cardari*, to 32.3% between *Aethionema grandiflorum* and *Lobularia maritima* var. *benthami*. The nonsynonymous to synonymous substitution ratio (Ka/Ks) was determined to be 0.0947.

According to the tribe assignment of Al-Shehbaz et al. [19], the 62 accessions belong to 14 tribes, with the remaining species *Lunaria annua* being unplaced. In the *FAE1* dataset, 6 tribes showed unique character states that differentiated them from the other tribes of Brassicaceae (Table S1). For example, tribes *Brassiceae* and *Lepidieae* display 16 and 2 diagnostic sites, respectively (e.g., positions 119: A and 506: C for *Brassiceae*; postions 284: G and 285: A for *Lepidieae*). Other tribes showing unique character states included *Cardamineae*, *Isatideae*, *Sisymbrieae* and *Thlaspideae*. A total of 12 diagnostic sites were also detected in 6 of the 31 genera, and 231 diagnostic sites were detected in 37 of the 62 species, with *Aethionema grandiflorum* exhibiting the greatest number of sites, at 53. One- and three-base-pair deletions (at positions 82 and 699-701) were found in the *FAE1* fragments from *Coronopus didymus* and *Aethionema grandiflorum*, respectively, leading to a frameshift mutation and premature termination of translation for the former and an amino acid reduction for the latter. 

### Phylogeny and grouping of *FAE1*

**Figure 1:**
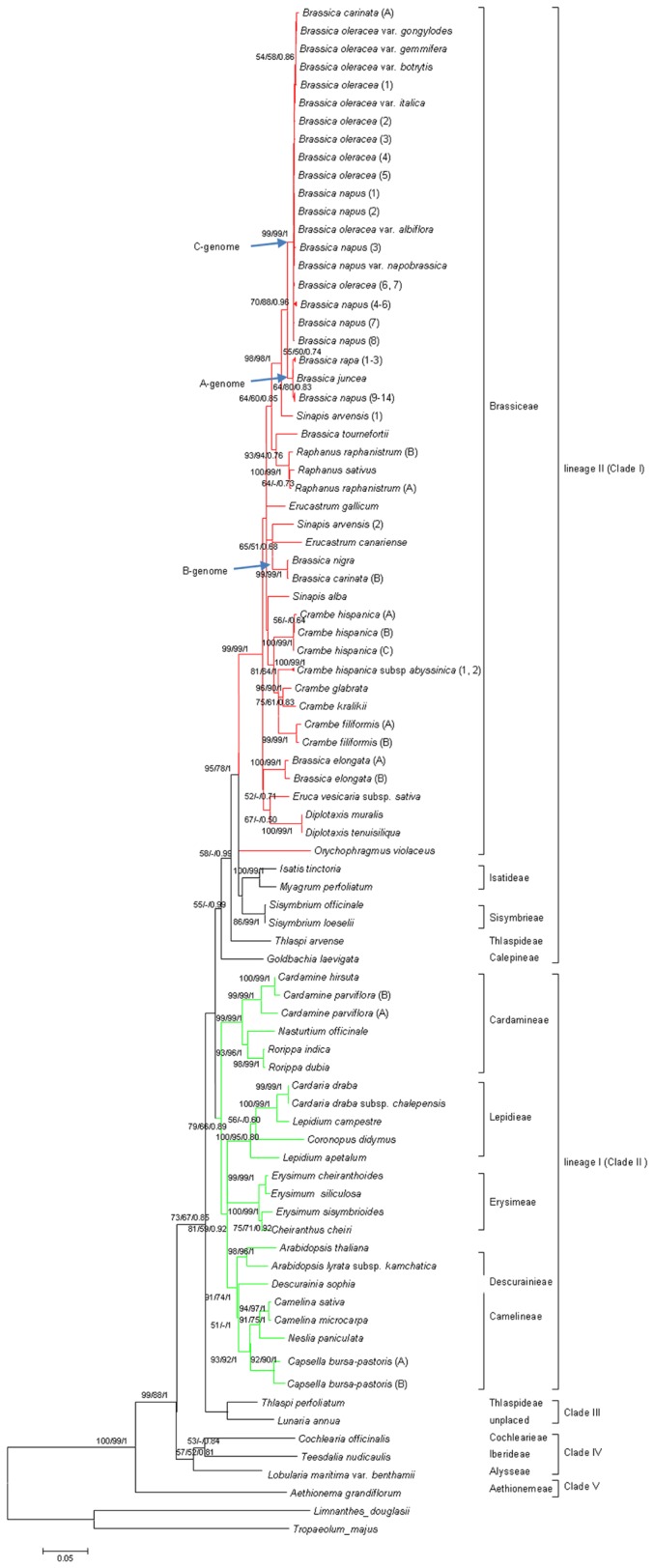
Concentrated Maximum likelihood phylogeny tree (-ln likelihood=30921.31) of Brassicaceae *FAE1* showing tribes. Numbers at branches are bootstrap percentages (the values less than 50% are not shown) of ML and MP and posterior probability of BI. Numbers or letters in brackets next to species represent different sequences downloaded from Genbank or different isoforms of the same species. See full Maximum likelihood phylogeny tree in Figure S1.

The phylogenetic tree of *FAE1* was obtained based on the maximum likelihood method (Figure 1; Figure S1). The parsimony and Bayesian inference trees displayed similar topologies to the maximum likelihood tree, which revealed a distinct grouping among the 62 accessions, with *Tropaeolum majus* and *Limnanthes douglasii* as outgroup. Five major clades were found: Clade V is sister to the remainder of the family, and the other clades (I-IV) are nearly equally distantly related. Bootstrap support for these clades was strong in some instances (e.g., greater than 55% for I, II, IV and V) but considerably weaker for Clade III (below 50%). Most of accessions in Clade I and all accessions in Clade II fall into lineage II and lineage I respectively, so these two clades hereinafter could be referred to as lineage II and lineage I respectively too. Within Clade I, 28 species of *Brassiceae* formed a strongly supported monophyletic group; other members of Clade I include two species each of *Sisymbrieae* and *Isatideae*, *Orychophragmus violaceus* and *Goldbachia laevigata*. The two species of *Thlaspideae* were separated in the *FAE1* gene tree: *Thlaspi arvense* and *Thlaspi perfoliatum* were distributed in Clades I and III, respectively. Clade II can be divided into two distinct sub-clades. Five species of *Cardamineae* formed a strongly supported sub-clade, and the species in the other sub-clade represent three tribes, *Camelineae*, *Erysimeae* and *Lepidieae*, together with one species each of *Descurainieae* and *Cardamineae*. *Thlaspi perfoliatum* and *Lunaria annua* formed a monophyletic Clade (III), though this placement showed moderate-weak support. Clade IV was well supported as monophyletic, including *Cochlearia officinalis, Teesdalia nudicaulis* and *Lobularia maritima*. Clade V was comprised of *Aethionema grandiflorum* and was strongly supported as a sister clade to the remainder of the Brassicaceae family.

The divergence levels (Dxy) between the five groups were calculated, revealing more than 13% of genetic divergence among them (Table 2). Group V was found to be considerably divergent from the other groups, and the nucleotide diversity (π) increased to 0.0679, 0.1063, 0.1920 and 0.2134 between group V and groups I, II, III and IV, respectively, with the diversity between alleles within groups V and IV showing extreme dissimilarity. Similarly, the diversity within a group decreased from 0.1715 in IV, 0.1110 in III and 0.0954 in II to 0.0626 in group I. Slightly different rates of diversity were obtained between the groups and closely related species within the same order (Brassicales). The lowest nucleotide diversity (0.0811) was found between group I and the out-group; group II differed from the out-group with an intermediate level of diversity (0.1383). The diversity values obtained between groups III, IV and V and the out-group were almost the same, at 0.3197, 0.3340 and 0.3671, respectively. The percentage of PS within a group reflects the richness of genetic variation in each group, and the obtained PS% values were 11.10%, 24.33%, 32.51% and 39.01% for groups III, IV, II and I, respectively. 

**Table 2:** Nucleotide diversity and divergence within and between groups (or families) and polymorphic sites at *FAE1*.

Group	Nucleotide diversity | divergence within or between groups					outgroup
	I	II	III	IV	V	
I	0.0626					0.0811|0.3863
II	0. 0927**|0.1336	0.0954				0.1383|0.3805
III	0.0667|0.1348	0.0996|0.1233	0.1110			0.3197|0.3726
IV	0.0721**|0.1759	0.1120**|0.1731	0.1649|0.1706	0.1715		0.3140|0.3845
V	0.0679|0.2466	0.1063|0.2295	0.1920|0.2323	0.2134|0.2546	-	0.3671|0.3926
Polymorphic sites (%)*	348(39.01%)	290(32.51 %)	99(11.10%)	217(24.33%)	-	

^**^ indicate a significant difference at P < 0.01 between two groups; the average nucleotide diversity between groups is calculated from all possible pairs between these groups; *Limnnthes douglasii* and *Tropaeolum majus* are used as out-group species.

^*^ indicates a significant difference (P < 0.001) between the numbers in different groups by the Chi-square test.

It is notable that the well-known allopolyploids in the genus *Brassica*, such as *Brassica napus*, produced two isoforms of the *FAE1* gene, with each clustering separately with its parental diploid species *Brassica oleracea* and *Brassica rapa*, corresponding to the C-genome of *Brassica oleracea* and the A-genome of *Brassica rapa*. The same situation was found in *Brassica carinata*, with the two isoforms corresponding to the C-genome of *Brassica oleracea* and the B-genome of *Brassica nigra*. Conversely, *Brassica juncea*, another allopolyploid, exhibited only one isoform clustered in the A-genome sub-clade because another isoform has not been identified and is therefore not included in this analysis. The divergence levels between the A-, B- and C-genomes showed more than 2% of genetic divergence between them, and 11, 45 and 47 mutations were detected as being fixed between the A- and C-genomes, B- and C-genomes or A- and B-genomes, respectively. The separate clustering of isoforms observed for each of *Crambe hispanica*, *Crambe hispanica* subsp *abyssinica*, *Crambe filiformis*, *Raphanus raphanistrum*, *Brassica elongata*, *Cardamine parviflora* and *Capsella bursa-pastoris* indicated that the *FAE1* genes of these species might have undergone a not particularly distant gene duplication or triplication event , perhaps along with or just after the species differentiation, similar to what has been observed for *Camelina sativa* [16]. 

### Relationship between the phenotypes and genotypes of *FAE1*

Our previous work determined the erucic acid contents in the seeds of 60 accessions [27], which ranged from 0 to 55.82% (Table 3). Gaps in the distribution of the erucic acid content, between 9.21% and 10.5%; 18.16% and 20.5%; 28.32% and 31.11%; and 39.8% and 41.1%, allowed us to group the alleles into five operational subclasses of erucic acid content in seeds, defined as low (L), intermediate (M1~M3), and high (H). This categorization of the accessions was strongly supported by an ANOVA analysis (*P* <<0.001 in a *t*-test for all pairs between the five categories). 

**Table 3:** Erucic acid content in seeds of 58 accessions in relation to genotypes of *FAE1*.

Accession	Erucic acid content ^1^	Category**	Fst between categories			
			M1	M2	M3	H
*Cardamine hirsuta*	0	L	0.0410	0.0940	0.2007	0.3977
*Cardamine parviflora*	0	L				
*Nasturtium officinale*	0	L				
*Lobularia maritima* var. *benthamii*	0.24	L				
*Orychophragmus violaceus*	0.37	L				
*Aethionema grandiflorum*	0.48	L				
*Capsella bursa-pastoris*	0.61	L				
*Coronopus didymus*	1.30	L				
*Arabidopsis thaliana*	1.88	L				
*Lepidium apetalum*	2.41	L				
*Camelina microcarpa*	2.53	L				
*Camelina sativa*	3.03	L				
*Goldbachia laevigata*	8.27	L				
*Arabidopsis lyrata* subsp. *kamchatica*	9.21	L				
*Neslia paniculata*	10.50	M1		0.0379	0.3255	0.3503
*Erysimum siliculosa*	11.01	M1				
*Cardaria draba*	11.04	M1				
*Cardaria draba* subsp. *chalepensis*	12.12	M1				
*Cochlearia officinalis*	13.77	M1				
*Sisymbrium loeselii*	16.89	M1				
*Sisymbrium officinale*	17.00	M1				
*Descurainia sophia*	18.16	M1				
*Erysimum cheiranthoides*	20.50	M2			0.2351	0.2598
*Erysimum sisymbrioides*	20.87	M2				
*Isatis tinctoria*	21.02	M2				
*Diplotaxis tenuisiliqua*	21.33	M2				
*Erucastrum gallicum*	21.91	M2				
*Rorippa indica*	22.32	M2				
*Lepidium campestre*	22.65	M2				
*Diplotaxis murali*	23.73	M2				
*Rorippa dubia*	26.16	M2				
*Myagrum perfoliatum*	28.32	M2				
*Sinapis alba*	31.11	M3				0.0218
*Erucastrum canariense*	31.53	M3				
*Cheiranthus cheiri*	31.62	M3				
*Sinapis arvensis*	34.51	M3				
*Brassica nigra*	34.88	M3				
*Raphanus sativus*	35.51	M3				
*Thlaspi perfoliatum*	36.19	M3				
*Brassica juncea*	36.96	M3				
*Thlaspi arvense*	37.79	M3				
*Raphanus raphanistrum*	39.79	M3				
*Brassica napus*	39.8	M3				
*Brassica oleracea*	41.10	H				
*Brassica carinata*	42.53	H				
*Eruca vesicaria* subsp. *sativa*	43.50	H				
*Brassica oleracea* var. *gemmifera*	44.00	H				
*Lunaria annua*	44.96	H				
*Crambe kralikii*	45.50	H				
*Brassica oleracea* var. *albiflora*	45.81	H				
*Brassica napus* var. *napobrassica*	46.41	H				
*Brassica elongata*	47.14	H				
*Brassica oleracea* var. *gongylodes*	47.94	H				
*Brassica oleracea* var. *botrytis*	48.03	H				
*Brassica tournefortii*	48.08	H				
*Brassica rapa*	48.32	H				
*Brassica oleracea* var. *italica*	49.77	H				
*Crambe filiformis*	51.88	H				
*Crambe hispanica* subsp *abyssinica*	53.12	H				
*Crambe hispanica*	55.82	H				
Overall Fst			0.2335*			

^1^ These data come from our previous work except that of *Crambe kralikii* and *Brassica napus* from Kumar and Tsunoda [47] and Velasco et al [48] respectively.

^*^ indicates a significant difference (P<0.01) for the genetic differentiation between different categories.

^**^ indicates a significant difference (P << 0.001) for each pairs between 5 categories by t-test.

The alleles from the low, intermediate and high erucic acid accessions were not scattered throughout the *FAE1* gene tree but grouped together (Figure 1). Therefore, we tested for a significant association between *FAE1* sequence variation and erucic acid content variation by analyzing the differentiation (an *F*st estimator based on nucleotide diversities [31]) between different phenotypes. The overall differentiation between the phenotypes was highly significant (L, M1, M2 and M3, H; Fst=0.2335, P=0.0062; Table 3), whereas no significant differentiation was detected between any of the pairs of phenotypes (Fst ≥0.0379, P ≥0.1168; Table 3). Thus, the sequence variation in the *FAE1* gene is correlated with the content of erucic acid in the seeds, which is suggestive of causal links between the genotype and phenotype.

**Figure 2:**
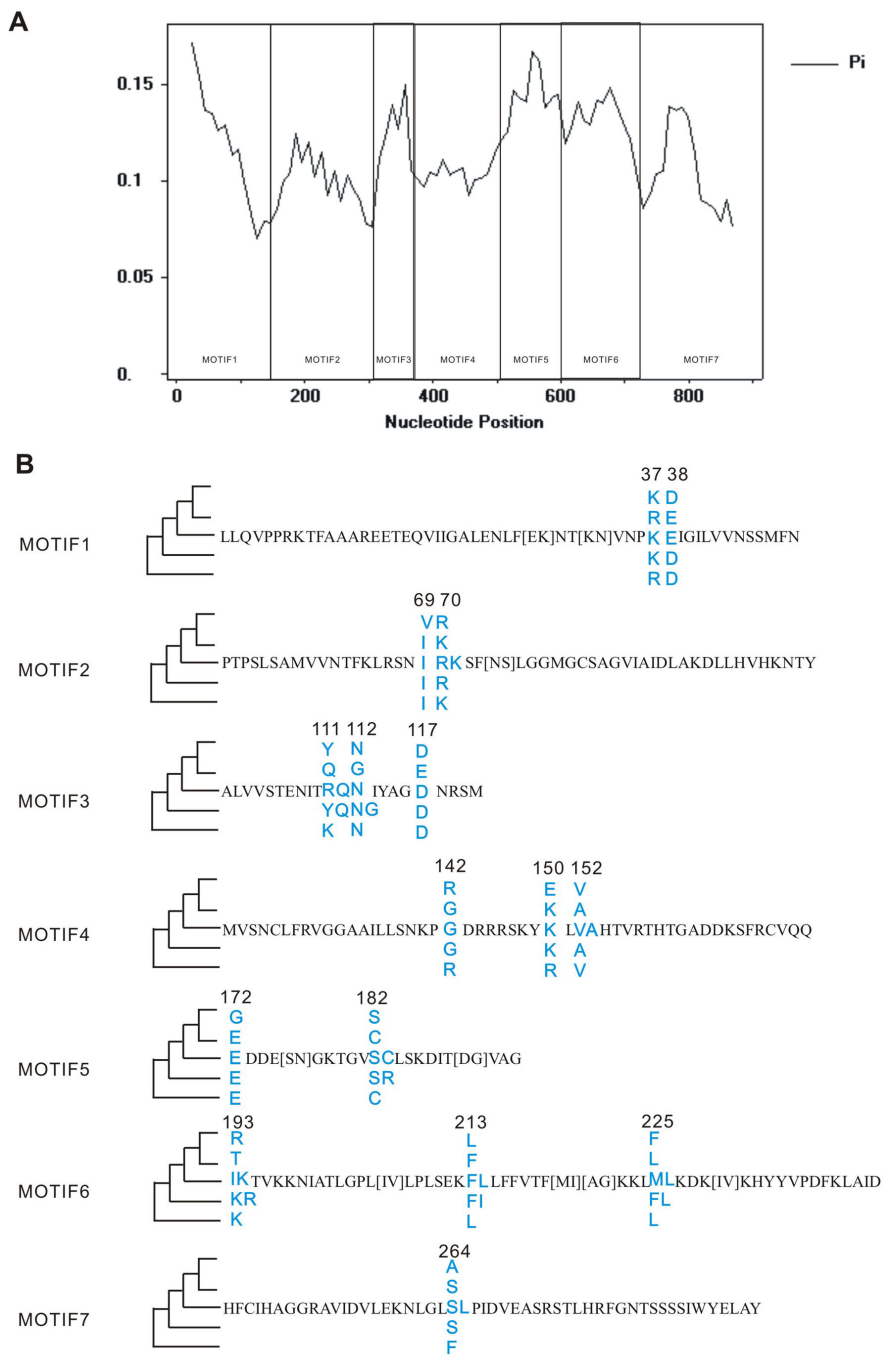
Sliding-window plots along the *FAE1* gene regions and fixed mutations between Clade I & II. (a) Diversity of the *FAE1* gene at silent sites. Windows include 25 silent sites, with successive displacements of 10 sites. The black boxes represent 7 conserved motifs, marked with corresponding ID. (b) 16 fixed mutations between Clade I & II in phylogeny tree of Brassicaceae *FAE1*. The consensus sequences of each conserved motif are shown with the simplified topology of phylogeny tree of Brassicaceae *FAE1* drafted in left. Blue characters in motifs indicate the fixed mutations and the variations of the fixed sites are listed corresponding to each clade in phylogeny tree.

To further explore the relationship between the observed genetic variation and phenotypes, the seven most conserved motifs within the *FAE1* coding sequence were identified using MEME (Figure 2). These motifs encompassed the entire *FAE1* encoding region, with no overlap being detected. Fixed mutations in one group reflect a conservative genetic state in that group that is unique from the other groups. Most of the accessions with high or low erucic acid contents were distributed in Clades I and II, and we detected 16 fixed mutations between these two groups (with a >70% allele frequency found in one group, but <30% in the other group), revealing the evolutionary history originating from the base (*Aethionema grandiflorum*) of Brassicaceae (Figure 2). In general, there were two indicated evolutionary pathways for a single mutation to have become fixed between the groups: one is to be diverged in Clades I and II from the existing alleles in the founder accessions (e.g., at positions 37, 38, 70, 111, 112, 142, 152, 182, 213 and 225); and the other is to newly appear in Clades I or II and differentiate itself from the other groups (e.g., at positions 69, 117, 150, 172, 193 and 264). Functional studies, such as through site-directed mutagenesis, could help to determine the active site(s) responsible for the function of *FAE1* enzyme.

## Discussion

### Brassicaceae phylogenetics inferred from *FAE1*

A comprehensive phylogeny of Brassicaceae based on the multiple plastid and nuclear genes revealed *Aethionema* as a sister group to the rest of the family (core Brassicaceae), which formed three well-defined major lineages (Lineages I-III), each consisting of several tribes [20-24,32]. The phylogenetic tree of the *FAE1* gene presented herein is largely congruent with a previous multiple gene-derived phylogeny, as follows. Clade I includes both tribes of Lineage II (Figure 1; with a well-support bootstrap value of 95) plus *Thlaspi arvense* (Thlaspideae) and *Goldbachia laevigata* (Calepineae), which have been considered to belong to in "expanded Lineage II" [31]; Clade II contains the tribes of Lineage I (Figure 1; tribes *Camelinae*, *Chorisporeae*, *Descuruainea*, *Erysimae*, *Cardamine*, *Lepideae*). *Thlaspi perfoliatum* (Thlaspideae) in Clade III and *Cochlearia officinalis* (Cochlearieae), *Teesdalia nudicaulis* (Iberideae) and *Lobularia maritima* var. *benthamii* (Alysseae) are placed in “expanded Lineage II” [31]. *Lunaria annua* in Clade III is not currently recognized in any of the three Lineages. The basal position of *Aethionema* is well supported by *FAE1* in this study and in previous reports [20-24,32].

In addition to the congruence of the five major clades with the well-recognized major lineages and basal groups, we report some further interesting results. For instance, *Descurainia* has been previously placed in the tribe *Sisymbrieae* [33]. Al-Shebaz et al. [17] reallocated this genus to a newly established tribe, *Descurainieae*, whereas *Descurainia* was later grouped with genera such as *Smelowskia* on the basis of multiple markers [21,24]. In this study, the separation of *Descurainia* from the *Sisymbrieae* tribe and the grouping of *Descurainia* within the clade of tribe *Camelineae* demonstrate the feasibility and rationality of the establishment of the new tribe *Descurainieae* and suggest a closer relationship of *Descurainia* with *Camelineae*. Additionally, the species within *Thlaspideae* were not grouped into the same clade but were dispersed in the phylogenetic trees obtained for the *PHYA* gene [21] and the *FAE1* gene in this study. These results suggest obvious differentiation in *Thlaspideae*, engendering the enthusiastic reconsideration of the previous taxonomy of *Thlaspideae*, which was mainly based on morphological characters.

### Selection on *FAE1*

The level of interspecific sequence diversity of the *FAE1* gene is clearly greater than that of chloroplast genes, though it is comparable to the levels found in some functional nuclear genes of Brassicaceae. The overall nucleotide diversity value of the nuclear-encoded chalcone synthase gene (*CHS*) reaches 0.1514% or 0.1373% with of without *Aethionema grandiflorum*, respectively [24]. Beilstein et al. [20,21] examined the levels of diversity in a chloroplast gene and a nuclear gene within a large sample of 113 species of Brassicaceae. The greatest sequence diversity detected in the chloroplast gene *ndhF* was 3.68% [20], whereas the sequence variation in nuclear phytochrome A (*PHYA*) was as high as 7.21% [21]. In contrast, a high level of polymorphism and a rapid rate of evolution have been detected in some other nuclear genes, such as plant disease resistance genes. Up to 9.8% nucleotide diversity has been reported for the *Rpp13* LRR region, even within populations of a single species [34]. Thus, an overall low level of nucleotide diversity (approximately 10%) together with the observed rates of nucleotide substitution in the *FAE1* gene indicate that this gene is under an evolutionary regime of purifying selective pressure, which is consistent with previous findings for another fatty acid elongase gene (Evovl5, a critical gene encoding an enzyme involved in long-chain polyunsaturated fatty acid (LC-PUFA) biosynthesis in fishes) [35].

### Differentiation of *FAE1* genotypes and phenotypes

In *Arabidopsis*, *FAE1* encodes for a b-ketoacyl-CoA synthase (*FAE1* KCS) that catalyzes the initial condensation step in the erucic acid elongation pathway [5]. Four conserved histidine residues (His302, -387, -391 and -420), six conserved cysteine residues (Cys84, -223, -270, -312, -389 and -460), including the active site at cysteine 223, and an asparagine residue at position 424, required for *FAE1* activity, were previously identified by Ghanevati and Jaworski [11,12]. All of these the residues were found to be conserved in the 96 Brassicaceae accessions, without substitution and are therefore not responsible for the observed alternations in contents of erucic acid in the different accessions. Although the overall differentiation analysis revealed strong causal links between *FAE1* sequence variation and seed erucic acid contents, the correlation for each pair of phenotypes was weak. A possible explanation for this result is that several alternative alleles of *FAE1* might confer the same activity, either resulting in the high or low trait. Both the high haplotypes and low haplotypes can be widely divergent; i.e., the *FAE1* protein can tolerate a significant number of substitutions while retaining its function, and there are many means of rendering an allele nonfunctional. As additional *FAE1* alleles are analyzed, it would be most interesting to identify alleles placed within a clade that lead to a high or low content of erucic acid and determine whether they are functional. One particular aspect of phenotypic variation is worth noting. *Orychophragmus violaceus* encodes an FAE1 protein that is highly similar to that of the other accessions in tribe *Brassiceae* (> 87% nucleotide identity), though the contents of erucic acid in the seeds of these accessions are substantially different. The seed erucic acid content of *Orychophragmus violaceus* is close to 0, whereas the *Brassiceae* accessions show contents as high as 21%~49%. Such phenotypic variation suggests that the two *FAE1* alleles are regulated differently. In support of this hypothesis, heterologous expression of the *FAE1* genes of *Orychophragmus violaceus* in yeast resulted in no erucic acid production, indicating that a loss of enzyme activity might be responsible for the low erucic acid trait in *Orychophragmus violaceus* [28]. 

Deletions in the *FAE1* sequence were proven to be responsible for the low erucic acid phynotype, corresponding to secondary mutations independent of point mutation [9,14,36]. A base-pair deletion in the *FAE1* sequence is first reported here in *Coronopus didymus*, which would lead to a frameshift mutation and a premature end of the translation. This one-base pair deletion was later shown to be one of the mutations responsible for the low erucic acid trait through heterologous expression in a yeast system [37] and is clearly independent of the previously reported two- and four-base-pair deletions [9,14,36]. Notably, there is another three-base-pair deletion (699-701) found at the base (*Aethionema grandiflorum*) of Brassicaceae. Interestingly, this three-base-pair deletion was also detected in all 32 whole-genome sequenced plants beginning with two members of the Chlorophyta, together with the two outgroup species in the same order, Brassicales. Therefore, this three-base-pair deletion is expected to represent an insertion occurring in the core Brassicaceae, exclusive of the base species. This deletion would introduce a tyrosine residue into the *FAE1-*encoded protein of all core Brassicaceae species. However, there have been no reports addressing the relationship between this tyrosine insertion and the function of the *FAE1* gene before, and based on the high levels of erucic acid observed in the two outgroup species in Brassicales, which do not exhibit tyrosine insertion, no strong evidence is provided of a correlation in the two outgroup species. Thus, it can only be concluded that this tyrosine insertion may serve as an indicator of a trend occurring during evolution from the base to the core of Brassicaceae.

In conclusion, the level of diversity and the available sequence data among different *FAE1* alleles provided a rich opportunity to study both evolutionary and genetic aspects of a known functional gene. Indeed, there is great potential for the utilization of evolutionary methods to evaluate diversity, combined with knowledge of gene structure and function to elucidate the molecular mechanisms underlying fatty acid synthesis. By determining the specific amino acid substitutions that alter the composition and content of specific fatty acids, inferences can be made about how the *FAE1* gene directs condensation within the elongation pathway of very long-chain fatty acids in plants. These functional studies can, in turn, help to define the reaction mechanism for a membrane-bound condensing enzyme.

## Materials and Methods

### Plant materials and orthologous sequences

Forty-eight accessions were collected from around the world, mostly from GRIN (Germplasm Resources Information Network) and PGRC (Plant Gene Resources of Canada) (Table 1). The seeds were geminated at the Institute of Botany, Jiangsu Province and the Chinese Academy of Sciences for DNA extraction. Orthologous sequences of *FAE1* from *Arabidopsis thaliana*, *Arabidopsis lyrata* subsp. *kamchatica*, *Brassica napus*, *Brassica rapa*, *Brassica oleracea*, *Crambe glabrata*, *Crambe kralikii*, *Crambe hispanica* subsp. *abyssinica*, *Isatis tinctoria*, *Lepidium apetalum*, *Orychophragmus violaceu, Sinapis alba*, *Sinapis arvensis* and *Teesdalia nudicaulis* were downloaded from GenBank (the GenBank accession numbers are provided in Table 1). In total, 62 accessions, representing 14 tribes, 31 genera, 51 species, 4 subspecies and 7 varieties were used in this study.

### *FAE1* cloning

For all materials, genomic DNA was extracted from fresh leaves using a modified cetyltrimethylammonium bromide (CTAB) method [38]. Four primer sets were selected from the literatures for *FAE1* gene amplification: TF, GCAATGACGTCCGTTAACGTTAAG, and TR, GGACCGACCGTTTTGGAC [29]; FAE1F, AGGATCCATACAAATACATCTC, and FAE1R, AGAGAAACATCGTAGCCATCA [28]; F, GCAATGACGTCCATTAACGTAAAG, and R, TTAGGACCGACCGTTTTGGGC [30]; B1F, ATCGGATCCATGACGTCCGTTAACGTAAAGCTCCTT, and B1R, ATCGAATTCTTAGGACCGACCGTTTTGGACA [14]. As to these samples failed to be amplified using the four primer sets, a new primer set (466S, AGATTCAAGAGCGTTCAGG, and 1497AS, TGACATTGCGTAGAGCCAC) was designed based on conserved regions with the aid of OLIGO primer design software (Molecular Biology Insights, Inc., Cascade, Colorado, USA) using the *FAE1*sequence of Brassicaceae deposited in the GenBank database. 

Polymerase chain reaction (PCR) amplification was performed using the following program: denaturation at 94°C for 3 min, followed by 35 cycles of denaturation at 94°C for 45 s, annealing at 53–58°C for 30 s and extension at 72°C for 1.5 min. Each 20-μl reaction mixture contained 30 ng of genomic DNA template, 2.5 mmol/L MgCl_2_, 1× Mg-free DNA polymerase buffer, 0.12 mmol/L dNTPs, 0.3 μmol/L each primer and 1 U of Taq DNA polymerase. The obtained PCR products were examined electrophoretically in 0.8-1.2% agarose gels. The purified products were sequenced either directly or after cloning into the pMD19-T vector if direct sequencing failed or dual peaks were found. At least three clones were sequenced for each collection. To exclude artificial singleton, the number of clones per individual was added until the haplotype was shared between at least two clones. As Brassicaceae includes several polyploidy species in which multiple copies of *FAE1* might exist [16,39,40], >20 colonies from 11 species randomly selected from 5 non-monospecies tribes, including 7 species of *Brassiceae* together with one each from *Calepineae*, *Cardamineae*, *Lepidieae* and *Thlaspideae*, were sequenced separately until no new homologous sequence could be identified. Bidirectional sequencing was completed by the Beijing Genomics Institute (BGI) using the same primers employed for amplification. 

### Sequence alignment and data analysis

The sequences were aligned and adjusted manually using Sequencer v.4.5 software (GeneCodes, Ann Arbor, MI, USA). and the nucleotide sequence data for the *FAE1* gene were deposited in the GenBank database (Table 1). All sequences, including those of two outgroup species in the same order (Brassicales), *Tropaeolum majus* L. (GenBank accession: AY082610) and *Limnanthes douglasii* R. Br. (GenBank accession: AF247134), were analyzed using MEGA (5.0 Version [41] and MrBayes (3.2.1 Version [42]) for phylogenetic reconstruction. The two methods of analysis applied in Mega included maximum likelihood (ML) and maximum parsimony (MP), with the bootstrap values being calculated from 1,000 replicates. The corrected Akaike Information Criterion in jModeltest [43] was used to identify the model of evolution that best fit the data for subsequent Bayesian inference analysis. We employed the DnaSp 4.0 program [44] to calculate population-genetic parameters, including the nucleotide diversity (π [45]), the average number of nucleotide differences per site between haplotypes (Dxy [45]), and the Ka/Ks ratio, to complete a sliding-window analysis, to determine silent and replacement sites. Without special explanation, indels were excluded in most calculations; otherwise, total number of polymorphic sites was increased by one site for each indel when length polymorphism sites were considered in these algorithms. 

The conserved motifs among the amino acid sequences of the *FAE1* region were identified and analyzed using MEME 4.6.1 (Multiple EM for Motif Elicitation [46]). Based on the expectation maximization, the conserved motifs were identified within a group of sequences without a priori assumptions about the alignments. The individual profiles for each conserved motif were assessed, and after tiling, only those conserved motifs showing P-values ≦ 10-4 and no overlap with each other were reported. 

## Supporting Information

Figure S1 **Full Maximum likelihood phylogeny tree (-ln likelihood=30921.31) of Brassicaceae *FAE1*.**
(TIF)

Table S1 **Diagnostic sites of tribe, genus and species.**
(XLS)
