# Supplementary figures and images for: Evolutionary Pattern of the FAE1 Gene in Brassicaceae and Its Correlation with the Erucic Acid Trait
Source: PLoS One. 2013 Dec 16;8(12):e83535. doi: 10.1371/journal.pone.0083535 (PMC3865303; doi:10.1371/journal.pone.0083535)

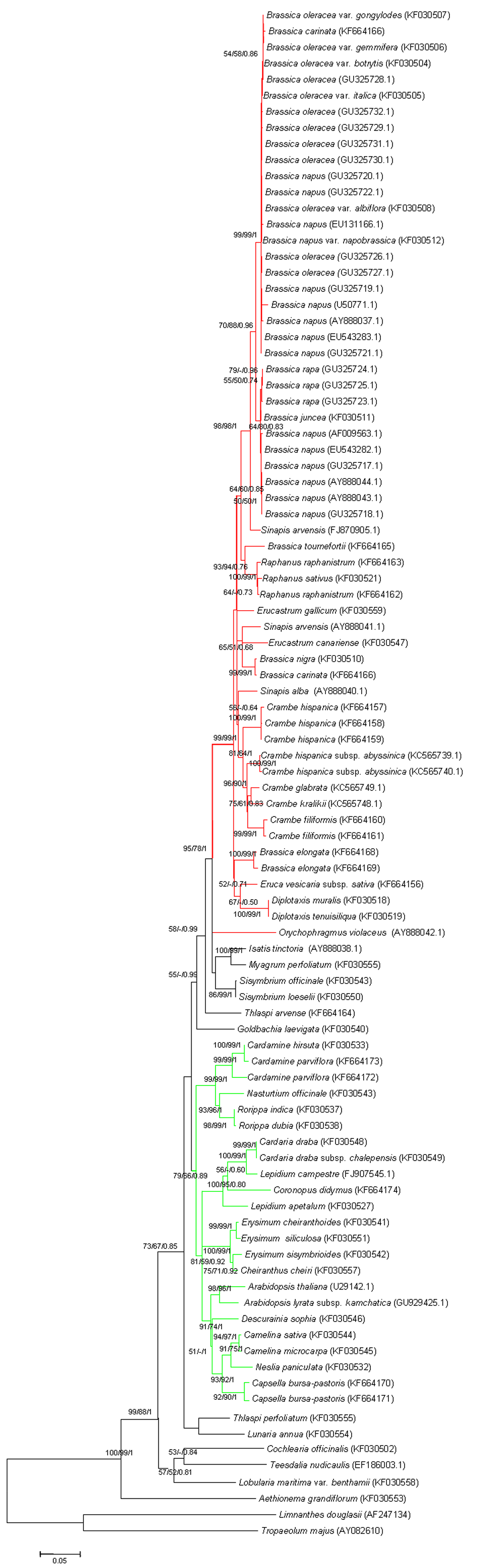

Supplement: Figure S1 — Full Maximum likelihood phylogeny tree (-ln likelihood=30921.31) of Brassicaceae FAE1. (TIF) [file pone.0083535.s001.tif]
